# Development of a scalable recombinant system for cyclic beta-1,2-glucans production

**DOI:** 10.1186/s12934-024-02407-z

**Published:** 2024-05-06

**Authors:** L. Soledad Guidolin, A. Josefina Caillava, Malena Landoni, Alicia S. Couto, Diego J. Comerci, Andrés E. Ciocchini

**Affiliations:** 1 Instituto de Investigaciones Biotecnológicas, Escuela de Bio y Nanotecnologías (EByN), Universidad Nacional de San Martín (UNSAM)-Consejo Nacional de Investigaciones Científicas y Técnicas (CONICET), Universidad Nacional de San Martín, San Martín, Buenos Aires, Argentina; 2 Centro de Investigación en Hidratos de Carbono (CIHIDECAR)- CONICET, Departamento de Química Orgánica, FCEN- Universidad de Buenos Aires (UBA), Pab. II, Ciudad Universitaria, Buenos Aires, Argentina

**Keywords:** Cyclic β-1,2-glucans, Cyclosophoraoses, Cyclodextrin, Oligosaccharides, Drug solubilization, Nanomaterial

## Abstract

**Background:**

Cyclic β-1,2-glucans (CβG) are bacterial cyclic homopolysaccharides with interesting biotechnological applications. These ring-shaped molecules have a hydrophilic surface that confers high solubility and a hydrophobic cavity able to include poorly soluble molecules. Several studies demonstrate that CβG and many derivatives can be applied in drug solubilization and stabilization, enantiomer separation, catalysis, synthesis of nanomaterials and even as immunomodulators, suggesting these molecules have great potential for their industrial and commercial exploitation. Nowadays, there is no method to produce CβG by chemical synthesis and bacteria that synthesize them are slow-growing or even pathogenic, which makes the scaling up of the process difficult and expensive. Therefore, scalable production and purification methods are needed to afford the demand and expand the repertoire of applications of CβG.

**Results:**

We present the production of CβG in specially designed *E. coli* strains by means of the deletion of intrinsic polysaccharide biosynthetic genes and the heterologous expression of enzymes involved in CβG synthesis, transport and succinilation. These strains produce different types of CβG: unsubstituted CβG, anionic CβG and CβG of high size. Unsubstituted CβG with a degree of polymerization of 17 to 24 glucoses were produced and secreted to the culture medium by one of the strains. Through high cell density culture (HCDC) of that strain we were able to produce 4,5 g of pure unsubstituted CβG /L in culture medium within 48 h culture.

**Conclusions:**

We have developed a new recombinant bacterial system for the synthesis of cyclic β-1,2-glucans, expanding the use of bacteria as a platform for the production of new polysaccharides with biotechnological applications. This new approach allowed us to produce CβG in *E. coli* with high yields and the highest volumetric productivity reported to date. We expect this new highly scalable system facilitates CβG availability for further research and the widespread use of these promising molecules across many application fields.

**Supplementary Information:**

The online version contains supplementary material available at 10.1186/s12934-024-02407-z.

## Background

Polysaccharides have important functions in all living systems and many biotechnological applications. To study their biological roles and properties and mainly to exploit their biotechnological applications, large amounts of pure polysaccharides produced at an affordable cost are required. Many polysaccharides are produced by bacteria and some of these polymers have been purified from the natural producers while others have been obtained by heterologous gene expression, particularly when the natural producers or the isolation and purification methods are not suitable for the required application [1–3].

Cyclic β-1,2 glucans (CβG) -also known as cyclosophoraoses (Cys)- are bacterial homopolysaccharides synthesized by members of the families *Rhizobiaceae* and *Brucellaceae* [4]. They occur in nature as a mixture of cyclic molecules formed by D-glucose as the unique sugar moiety with a degree of polymerization (DP) ranging from 17 to 25 units, although some *Rhizobium* glucans can reach up to 40 units. These molecules are exclusively linked by β-1,2 linkages and can be unsubstituted or decorated with phosphoglycerol, succinyl or malonyl moieties or a combination of them depending on the bacterial species. The absence of a reducing end and the type of glycosidic bond (β-1,2) turns the CβG into highly stable molecules [5]. Due to their ring shape and chemical properties, CβG resemble cyclodextrins which are molecular containers capable of entrapping, solubilizing and stabilizing a variety of hydrophobic guest compounds, among many other interesting properties [6]. Native cyclodextrins are considered natural products in Japan allowing their use in both the pharmaceutical and food industries with few restrictions. The ingestion of native cyclodextrin is “Generally Regarded as Safe” by the FDA (GRN678), although certain limitations apply to their parenteral use [6]. After more than 100 years of research, the FDA approval of cyclodextrins and their derivatives enhanced their applications and boosted the market growth for these molecules. Now they can be found in multiple pharmaceutical, cosmetic and food products [7]. Like cyclodextrins, CβG have a great industrial potential due to their chemical and immunological properties. Fields of applications include pharmaceuticals (drug solubilization, immunomodulation), medical technology (dye binding), chemistry (enantiomer separation, catalysis, click chemistry), food and nanomaterials among others [5, 8–10].

Despite being promising molecules, the biological functions and biotechnological applications of CβG have not been extensively investigated. One of the reasons for this is the inability to chemically synthesize CβG and the fact that most CβG-synthesizing bacteria are pathogenic or require long incubation times to produce CβG, resulting in low productivity. Furthermore, the properties of the CβG obtained, such as size, identity and the number of substituents per molecule are dependent on the specific microorganism and culture conditions [5]. Early reports on CβG synthesis indicated yields ranging from milligrams to a few grams per liter of culture, producing a mixture of neutral and substituted CβG and often in complex mixtures with other polysaccharides that hinder the downstream processes [2, 11–14]. Mutagenesis of natural producer strains, optimization of the culture medium composition and the incubation temperature redirected polysaccharide synthesis and/or increased the yield of CβG up to 5–10 g/L in certain *Agrobacterium* and *Rhizobium* species. However, these higher yields were achieved with extended incubation times of 8 to 15 days in nitrogen-limited cultures, resulting in low overall productivities [13, 15, 16].

In recent years, the biochemistry, genetics and structural biology underlying the bacterial biosynthesis of CβG has been described in detail [5, 17]. These molecules are produced by a protein complex of three polytopic inner membrane proteins, the CβG synthase (Cgs) the CβG transporter (Cgt) and the CβG modifier (Cgm) [18]. Cgs is a multimodular protein of 320 kDa that acts as a protein-sugar intermediate and catalyzes the four enzymatic reactions required for CβG synthesis: initiation, elongation, phosphorolysis, and cyclization [19, 20]. After CβG are released from Cgs into the cytoplasm, Cgt transports the CβG to the periplasmic space [21] and, once in the periplasm, CβG are decorated with succinyl residues by Cgm conferring an anionic character to the molecules [22]. Based on this knowledge, it is possible to devise a recombinant system that allows for the scalable production of CβG for biotechnological applications.

In this work, we have engineered an *Escherichia coli* strain as a platform for the synthesis of CβG by expression of the genes involved in the biosynthesis of these cyclic polysaccharides. In this way, we were able to produce neutral unsubstituted CβG, anionic CβG and CβG with high DP. Additionally, this platform allowed us to scale up the production of unsubstituted CβG with high yields and increased productivity by High Cell Density Culture (HCDC) of *E. coli*.

## Results

### Synthesis and secretion of CβG in *E. coli*

**Fig. 1:**
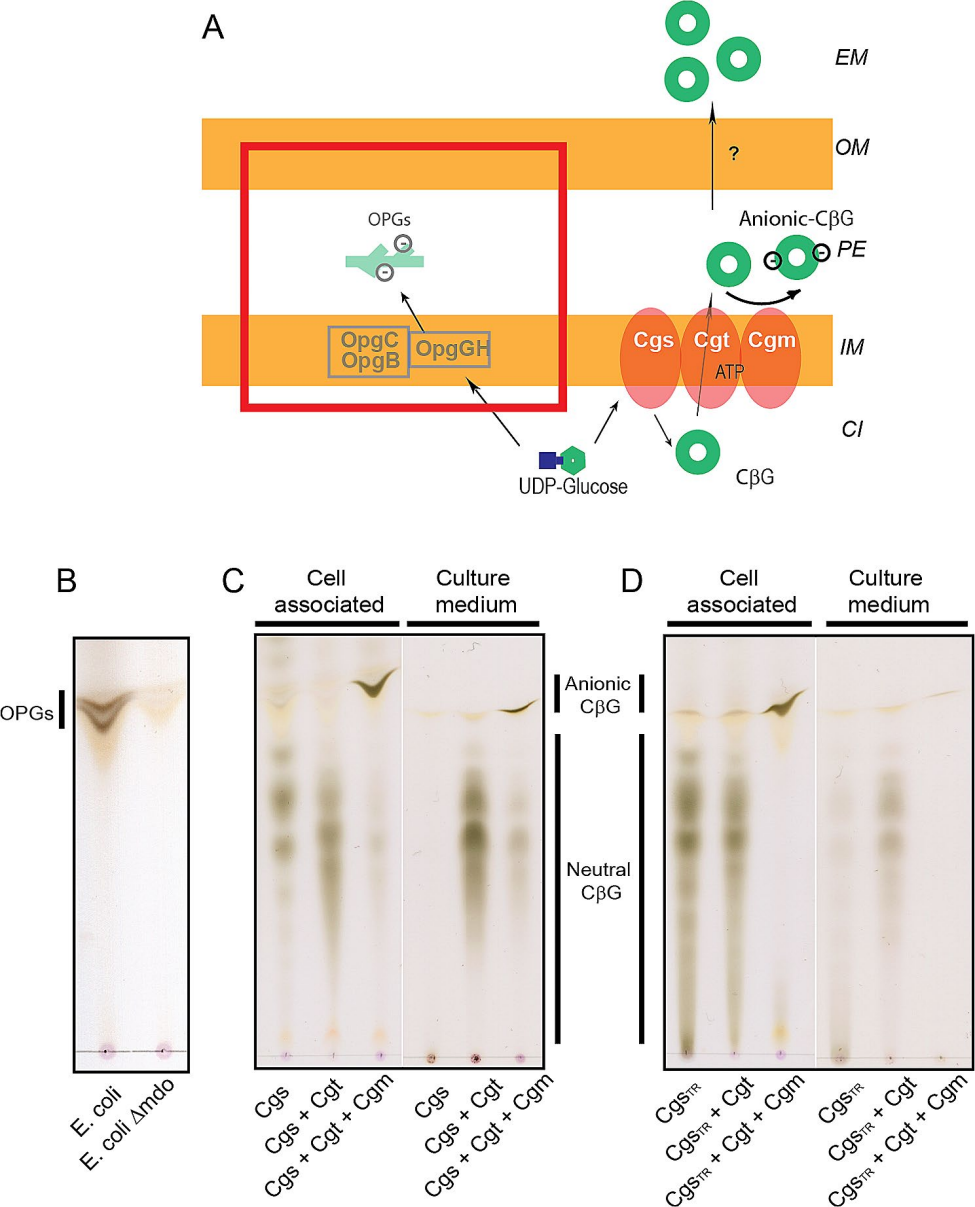
Cyclic β-1,2-glucans (CβG) produced in *E. coli*. (**A**) Schematic representation of *E. coli Δmdo* strain expressing Cgs, Cgt and Cgm to produce CβG. EM, extracellular medium; OM, outer membrane; PE, periplasm; IM, inner membrane; CI, cytoplasm; CβG, Cyclic β-1,2-glucans; OPGs, Osmoregulated periplasmic glucans or β-1,6-branched-β-1,2-lineal glucans. (**B**) TLC analysis of ethanolic extracts from parental *E. coli* strain or *Δmdo* mutant (*ΔmdoGH ΔmdoC ΔmdoB*). Osmoregulated periplasmic glucans (OPGs) or anionic β-1,6-branched-β-1,2-lineal glucans of *E. coli* are highly decorated with anionic substituents giving sharp bands in this TLC system. The *E. coli Δmdo* mutant lacks OPGs. (**C**) TLC analysis of cellular and culture-medium fractions of CβG produced in *E. coli Δmdo* expressing Cgs (*E. coli Δmdo*^Cgs^); Cgs and Cgt (*E. coli Δmdo*^Cgs+Cgt^) or Cgs, Cgt and Cgm (*E. coli Δmdo*^Cgs+Cgt+Cgm^). *E. coli Δmdo*^Cgs^ and *E. coli Δmdo*^Cgs+Cgt^ stains produce cell-associated unsubstituted CβG, which can be obtained from cells by ethanol extraction (left). Unsubstituted CβG produced by the strains *E. coli Δmdo*^Cgs+Cgt^ and *E. coli Δmdo*^Cgs+Cgt+Cgm^ reach the extracellular space and can be recovered from the culture medium supernatant (right). *E. coli Δmdo*^Cgs+Cgt+Cgm^ strain also produces anionic CβG that are mostly retained in the cell fraction. (**D**) TLC analysis of cellular and culture-medium fractions of CβG produced in *E. coli Δmdo* expressing Cgs mutant truncated in the phosphorylase domain (Cgs_tr_, stain *E. coli Δmdo*^Cgstr^); Cgs_tr_ and Cgt (*E. coli Δmdo*^Cgstr+Cgt^) or Cgs_tr_, Cgt, and Cgm (*E. coli Δmdo*^Cgstr+Cgt+Cgm^). Expression of Cgs_tr_ resulted in the production of CβG with a reduced mobility in the TLC, indicative of a higher degree of polymerization. Unlike CβG with normal size, these bigger CβG are mostly retained in the cell fraction

CβG are synthesized by a membrane protein complex formed by Cgs, Cgt and Cgm. This complex is necessary and sufficient for the synthesis of CβG and is active when expressed in the *E. coli Δmdo* strain [18]. *E. coli Δmdo* mutant strain has a deletion in the *mdoGH* operon and the *mdoB* and *mdoC* genes which are responsible for the synthesis and substitution of β-1,6-branched β-1,2-linear glucans, also known as osmoregulated periplasmic glucans (OPGs) (Fig. 1A). *E. coli Δmdo* lacks OPGs (Fig. 1B) allowing thin layer chromatography (TLC) analysis of CβG (OPGs migrates at the same position than charged CβG) and simplifying its purification. Furthermore, since CβG and OPG biosynthesis depend on the availability of the sugar donor UDP-Glucose, deletion of OPG genes is expected to improve CβG production.

To analyze the production of CβG in *E. coli Δmdo*, we constructed three strains: *E. coli Δmdo*^Cgs^ strain expressing the *B. abortus* CβG synthase (Cgs), *E. coli Δmdo*^Cgs+Cgt^ expressing Cgs and the CβG transporter (Cgt), and *E. coli Δmdo*^Cgs+Cgt+Cgm^ which expresses Cgs, Cgt and the CβG modifier Cgm. All three and control strains were grown in M9 saline defined medium with glycerol as sole carbon source. For the analysis of CβG production, cultures were harvested by centrifugation and CβG in the cell pellets and culture supernatants were analyzed by TLC as described in Methods. We observed that expression of Cgs in the *E. coli Δmdo*^Cgs^ and *E. coli Δmdo*^Cgs+Cgt^ strains resulted in the synthesis of neutral and cell-associated CβG (Fig. 1C, lane 1 and 4). In addition, co-expression of Cgs and Cgt in *E. coli Δmdo*^Cgs+Cgt^ resulted in a higher production of CβG and the secretion of CβG into the culture medium (Fig. 1C, lane 2 and 5). When Cgs, Cgt and Cgm proteins were co-expressed in *E. coli Δmdo*^Cgs+Cgt+Cgm^, most of the CβG became anionic and retained in the cell fraction (Fig. 1C, lane 3 and 6). The anionic fraction of CβG turned into neutral CβG after mild-alkali treatment (data not shown), consistent with the O-ester substitution of the glucans with succinyl residues.

We have previously described that the DP of CβG is controlled by the C-terminal phosphorylase domain of Cgs [19]. As the size of the CβG could change their biochemical properties and biotechnological applications, a Cgs truncated protein with a deletion in the C-terminal phosphorylase domain (Cgs_tr_) was expressed in *E. coli Δmdo*. As shown in Fig. 1D, expression of Cgs_tr_ in *E. coli Δmdo*^Cgstr+Cgt^ strain resulted in the production of CβG with a reduced mobility in the analysis by TLC, indicative of the production of molecules with a higher size. Those CβG with high DP do not seem to be secreted to the culture medium in *E. coli Δmdo*^Cgstr+Cgt^. Furthermore, *E. coli Δmdo*^Cgstr+Cgt+Cgm^ also produced anionic CβG which were all retained in the cell fraction (Fig. 1D).

In summary, we were able to develop a recombinant system in *E. coli* that produces different kinds of CβG in a simple and reproducible manner in a safe and fast-growing host. This platform allows uncoupling CβG synthesis from substitution and the synthesis of neutral and anionic CβG as well as CβG with a higher DP. Furthermore, in *E. coli Δmdo*^Cgs+Cgt^ much of the unsubstituted CβG were secreted into the culture medium which could be an advantage for scaling up the production and purification of these molecules; therefore, we decided to focus on the optimization of the production process using the *E. coli Δmdo*^Cgs+Cgt^ strain.

### Production of unsubstituted CβG in shake flask experiments

**Fig. 2:**
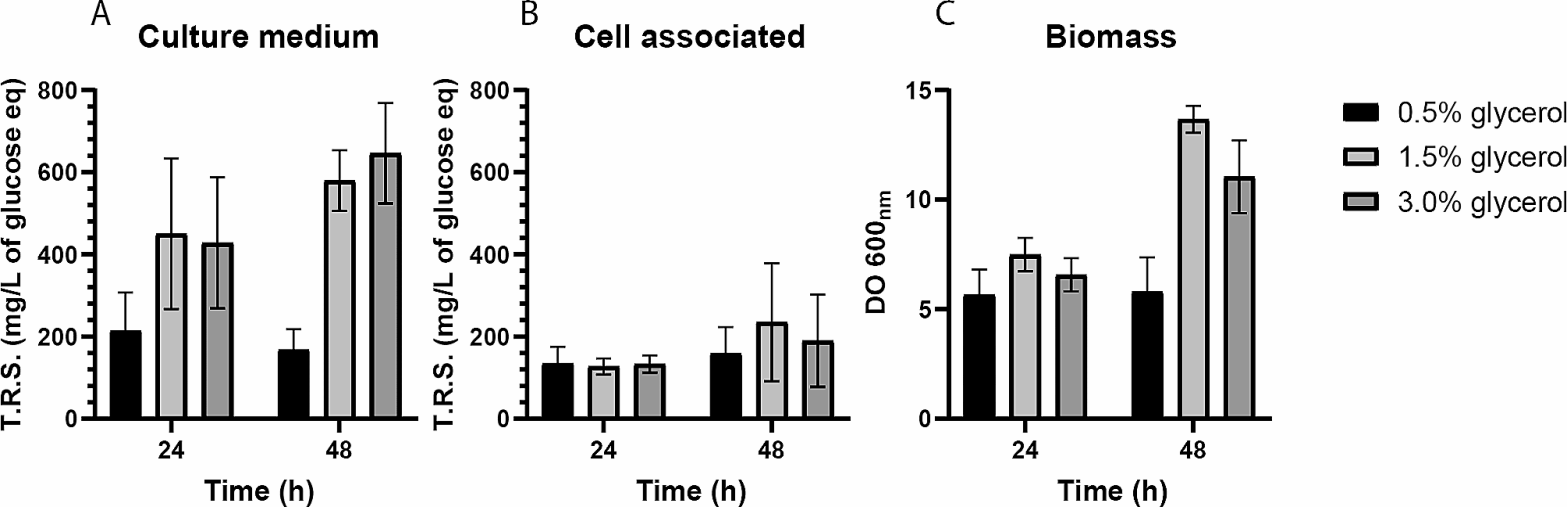
CβG and biomass production at different culture times and substrate concentrations. (**A**) Production of CβG in the culture supernatant. (**B**) Production of cell-associated CβG. (**C**) Biomass production. *E. coli Δmdo*^Cgs + Cgt^ strain was grown for 48 h in culture media containing 0.5%, 1.5% or 3% glycerol as carbon source. OD at 600 nm was measured after 24 and 48 h of incubation at 30 °C and agitation at 200 rpm. CβG were extracted from the cells or precipitated from the culture supernatant with ethanol and the total reducing sugars (TRS) content was measured by the anthrone-sulfuric acid method. CβG levels were expressed as mg/L glucose equivalents. Error bars represent the standard deviation of three independent experiments

In order to determine the best configuration for the production of unsubstituted CβG in *E. coli Δmdo*^Cgs+Cgt^ strain, several variables of the culture conditions were analyzed in shake flask experiments. Cultures were performed in Korz saline defined medium [23] with glycerol as carbon source. Glycerol was chosen as carbon source because the *cgs* and *cgt* genes are under the control of the *lac* promoter and therefore its expression is inhibited by glucose (catabolic repression). We have previously observed that Cgs expression in *E. coli* is better at 30 °C than at 37 °C [18], so the growth temperature was set at 30 °C. The influence of substrate concentration on biomass and CβG production was analyzed by measuring the OD at 600 nm and total reducing sugars (TRS) since most of the reducing sugars in the culture supernatant and ethanolic cell extracts correspond to CβG. Biomass and TRS accumulation in the cell pellet and supernatant were analyzed at 24 and 48 h of incubation and for all the conditions, a high accumulation of TRS in the culture medium was observed (Fig. 2). Cultures with 0.5% glycerol were clearly limited in carbon source and reached the maximum biomass and TRS at 24 h of incubation. Instead, cultures with 1.5 and 3% glycerol continued to grow and produced similar amounts of TRS at 24 and 48 h of incubation, while biomass showed a slight degree of inhibition with 3% glycerol (Fig. 2). We also observed that nitrogen-limited cultures with an excess of glycerol did not increase the production of CβG (data not shown) as described in rhizobia [13, 16]. These results indicate that there is a correlation between the increase in biomass and CβG production.

**Fig. 3:**
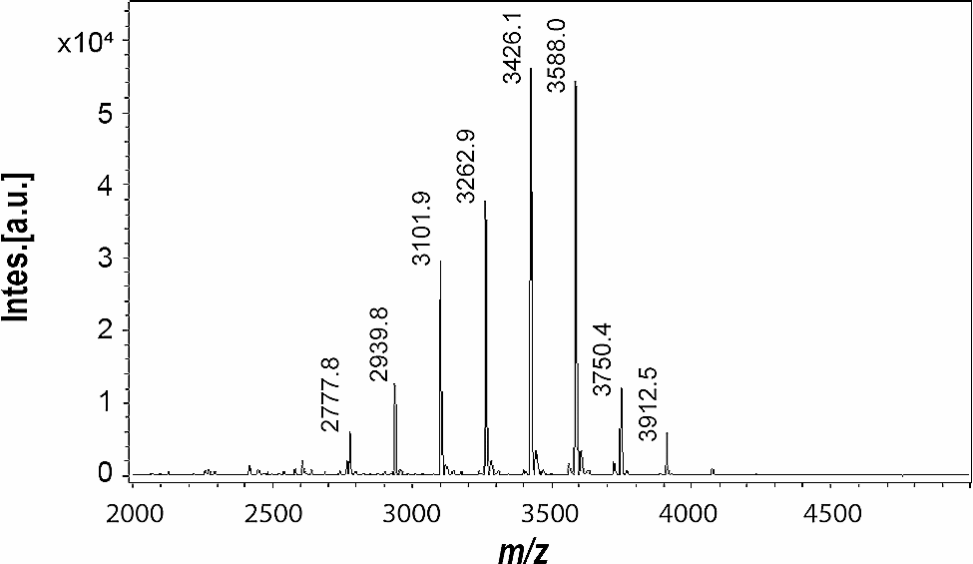
MALDI-TOF mass spectrometry analysis of CβG produced by *E. coli Δmdo*^Cgs+Cgt^ in Erlenmeyer and purified from culture supernatant. Signals with *m/z* corresponding to cyclic molecules from 17 to 24 glucose units were detected

To characterize the CβG secreted by the *E. coli Δmdo*^Cgs+Cgt^ strain, CβG were purified by ethanol precipitation and size exclusion chromatography (SEC) and analyzed by MALDI-TOF mass spectrometry. Analysis of the spectrum revealed nine main signals at *m/z* values of 2777,8; 2939,8; 3101,9; 3262,9; 3426,1; 3588,0; 3750,4, and 3912,5 which are consistent with unsubstituted cyclic glucan molecules with a DP of 17 to 24 glucoses, being those with a DP of 19 to 22 DP the main species (Fig. 3), a similar size distribution than observed in *Brucella* [10].

### Production of unsubstituted CβG in high cell density culture (HCDC)

Previous results indicate a correlation between the increase in biomass and CβG production. To maximize these parameters, a HCDC of *E. coli Δmdo*^Cgs+Cgt^ in a stirred tank bioreactor was performed. The kinetic and stoichiometric constants for designing the bioreactor culture were first calculated in shake flask experiments (Erlenmeyer). We observed that biomass accumulation followed an exponential behavior with a maximum growth rate (µ_max_) of 0.19 h^− 1^ (doubling time = 3.6 h). The equivalence of dry cell weight (dcw) was 0.3 g dcw/L of cells at OD_600nm_ = 1, and the biomass yield (Y_x/s_) was 0.37 g_x_/g_glycerol_ (Figure S1).

**Fig. 4:**
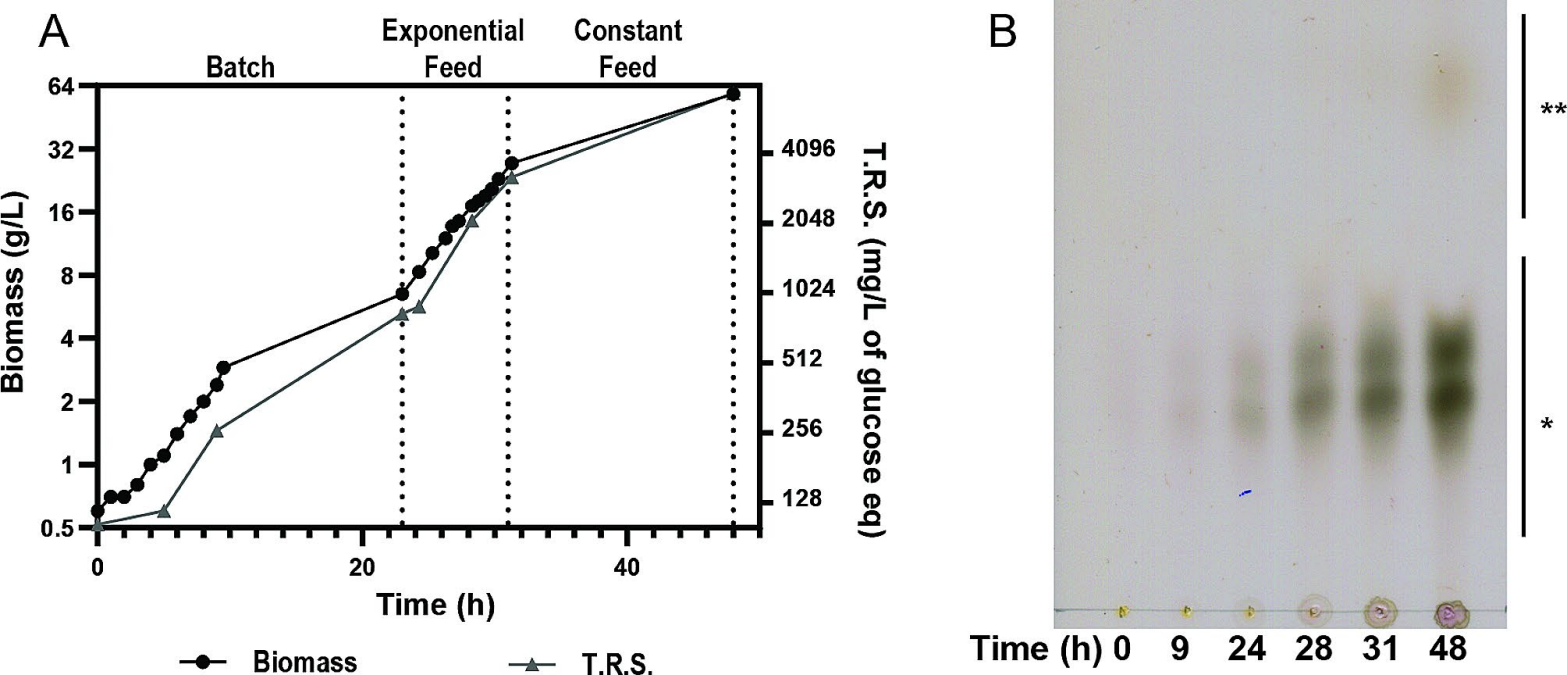
CβG production in High Cell Density Culture (HCDC) of *E. coli Δmdo*^Cgs+Cgt^. (**A**) HCDC was performed on Korz medium with glycerol as carbon source and consisted of 3 steps: an initial batch step, an exponential feed batch step performed to increase biomass until oxygen transfer became the limiting factor, and a constant feed regime to reduce oxygen demand until 48 h of effective fermentation time (EFT). Biomass production was followed by DO_600nm_ and dry weight determination. CβG production was indirectly followed by measurement of total reducing sugars (TRS) in the culture supernatant and expressed as g/L glucose equivalents. (**B**) Analysis of CβG production by TLC. Equivalent volumes of culture supernatant purified by ethanol precipitation were analyzed. The results are representative of two independent experiments of HCDC. *, migration position of unsubstituted CβG; **, migration position of non-CβG sugar products

HCDC was performed in three stages: an initial batch step with 10 g/L glycerol to obtain biomass, an exponential fed-batch phase with a growth rate of 0.16–0.2 h^− 1^ until oxygen uptake became the limiting factor, and a constant (30 ml/h) fed-batch step in order to obtain a fully aerobic culture and complete biomass accumulation up to 48 h of effective fermentation time (EFT). At this final stage, the process was stopped, and the culture was harvested (Fig. 4A). At different incubation times, CβG were quantified indirectly by measuring TRS and characterized by TLC (Fig. 4A and B). We observed that CβG accumulation achieved the same kinetic profile as biomass, indicating that CβG production is effectively associated with bacterial growth (Fig. 4A).

**Fig. 5:**
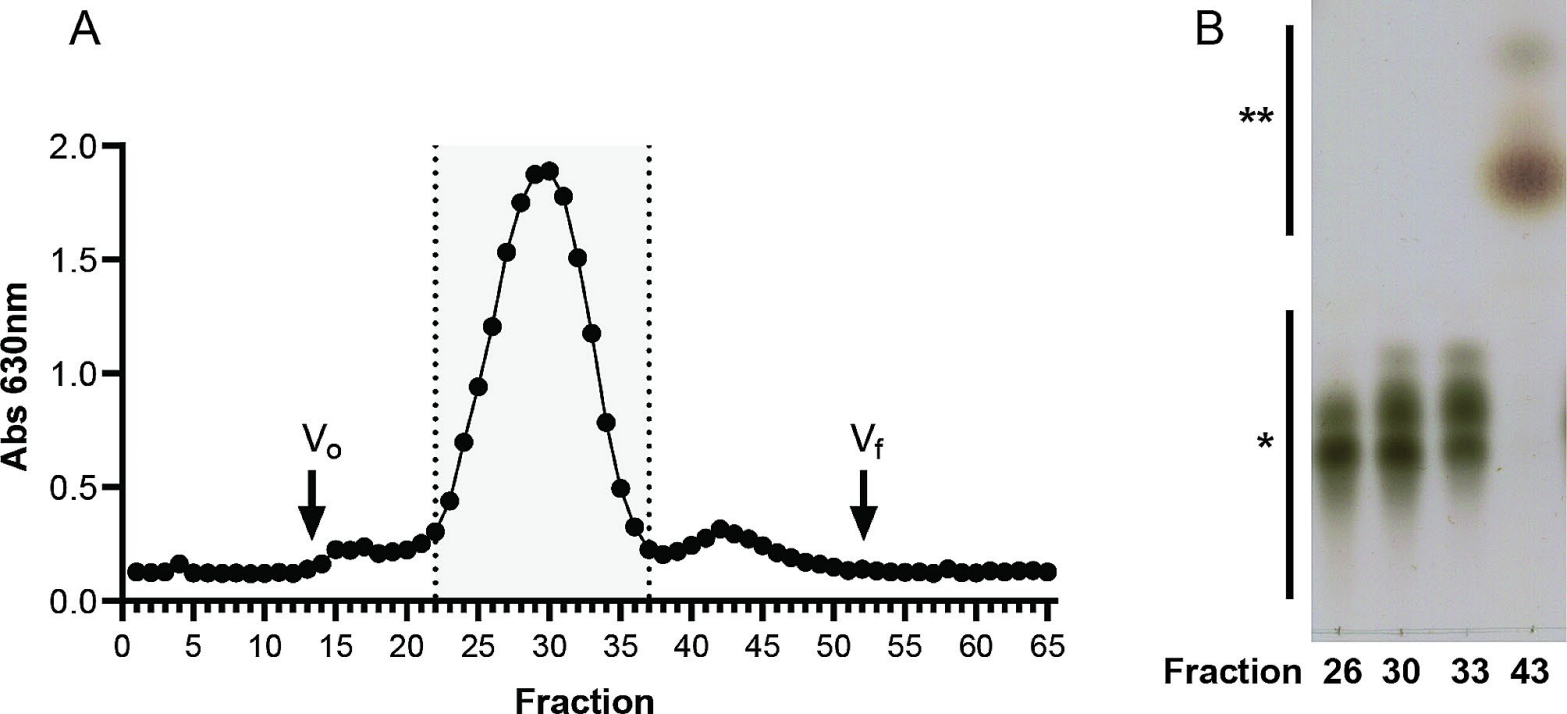
Purification and characterization of CβG produced by HCDC of the *E. coli Δmdo*^Cgs+Cgt^. (**A**) Purification of CβG by size exclusion chromatography (SEC). A small volume of HCDC culture supernatant was subjected to SEC on a BioGel P6 column (16/170 mm) and the resulting fractions were quantified by the anthrone-sulfuric acid method. (**B**) Characterization of CβG by TLC. Representative samples of each peak were subjected to qualitative analysis by TLC. *, migration position of unsubstituted CβG; **, migration position of non-CβG sugar products

**Fig. 6:**
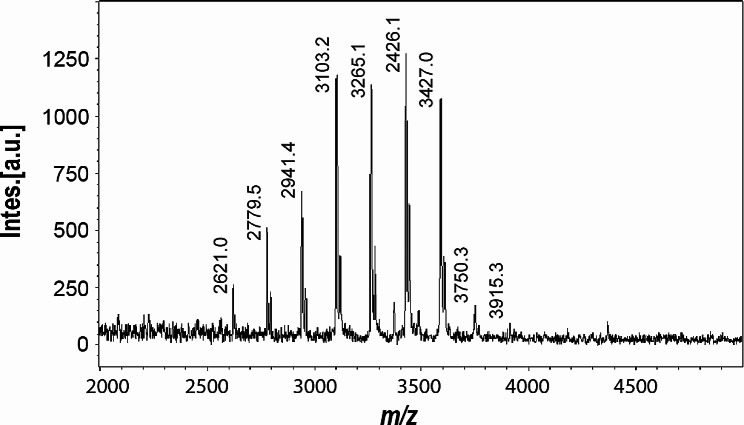
MALDI-TOF mass spectrometry analysis of CβG produced by *E. coli Δmdo*^Cgs+Cgt^ in stirred tank bioreactor and purified from culture supernatant. Signals with *m/z* corresponding to cyclic molecules from 16 to 24 glucose units were detected

After 48 h of EFT of the HCDC, the entire culture was harvested by centrifugation and a fraction of the culture media was subjected to SEC to purify CβG and calculate the yield of the purified product (Fig. 5A). Column fractions were analyzed by the anthrone-sulfuric acid method and representative positive fractions were characterized by TLC analysis (Fig. 5B). Only anthrone-positive fractions of the SEC column corresponding to CβG were considered to quantify the final yield of purified CβG. CβG produced in stirred tank bioreactor were characterized by MALDI-TOF-MS (Fig. 6). The observed m/z signals correspond to unsubstituted CβG with 16 to 24 glucoses, where the species of 19 to 22 glucoses were the most abundant and distributed in similar proportions.

As a result of two independent experiments, we obtained an average of 4.5 g of purified CβG/L in the culture supernatant after 48 h of incubation (7.1 g T.R.S./L of crude supernatant), with a yield (Y_p/s_) of 41 mg CβG/g_glycerol_ and an intrinsic productivity (Y_p/x_) of 100 mg CβG/g_dcw_ (Table 1). This corresponds to 94 mg L^− 1^h^− 1^ of CβG, the highest volumetric productivity reported to our knowledge.

**Table 1:** Parameters obtained in two HCDC of the *E. coli* Dmdo^Cgs+Cgt^strain

Parameter	1	2	Avg ^a^
Purified CbG (g/L)	4,4	4,6	4,5
Crude T.R.S.^b^ (g/L)	7,4	6,7	7,1
Final Biomass (g)	109	117	113
Glycerol (g)	249	305	277
µ_fedbach_ (h^− 1^)	0,16	0,21	0,18
Y_x/s_ (g _dcw_/g_gli_)	0,44	0,38	0,41
Y_p/s_ (mg_CbG_/g_gli_)	38	44	41
Y_p/x_ (mg_CbG_/g_dcw_)	87	115	101
t (h)	48	48	48
Vol. prod. (mg/Lh) ^c^	92	96	94

^a^ Avg: average value of the parameters for the two experiments

^b^ T.R.S. in culture medium

^c^ Vol. prod.: volumetric productivity

## Discussion

In this work, we have developed a new platform to produce CβG in *E. coli*, a well-established, safe and fast-growing host. A variety of carbohydrate structures ranging from small oligosaccharides to high molecular weight polymers have been produced or transformed by bacterial expression systems. Examples of them are milk oligosaccharides, hyaluronate, alginates, capsular polysaccharides (CPS from *Neisseria meningitidis*, *E. coli* K1, *Staphylococcus* and *Streptococcus*) and glycoconjugates [1, 3]. In particular, multiple α- and β-glucans like cellulose, dextran, curdlan and cyclic α- and β-glucan, among others, have been produced in bacteria [2]. Compared to other polysaccharides, CβG have the advantage of not requiring a primer or acceptor substrate for its synthesis since Cgs acts as an autoglucosyltransferase that primes itself and autocatalytically initiates the synthesis of CβG. In addition, CβG is an homopolysaccharide linked by a unique type of glycosidic linkage (β-1,2) making its synthesis relatively simple compared to other heteropolysaccharides [5]. In our system, an engineered *E. coli* strain that lacks OPGs allows the production of different types of CβG through the selective expression of the enzymes involved in biosynthesis (Cgs), transport (Cgt) and modification (Cgm) of CβG. This approach allows uncoupling CβG synthesis from the modification of these molecules with succinyl residues and enables the synthesis of CβG with a high DP. As a result, we have obtained homogeneous batches of CβG with reduced contamination by other CβG variants and polysaccharides, simplifying the downstream purification process. In addition, this platform exhibited high yields for the synthesis of unsubstituted CβG by HCDC of *E. coli*.

CβG can be used unsubstituted or can be chemically-modified for different applications as it is currently done with cyclodextrins. Similar to cyclodextrins, CβG and their derivatives find applications across multiple fields. The most evident application lies in their ability to encapsulate a wide range of insoluble compounds. For instance, they can encapsulate nonsteroidal anti-inflammatory drugs like ibuprofen, naproxen and indomethacin. Neuroleptic substances such as propericiazine, glucocorticoids like dexamethasone, antitumoral drugs like paclitaxel, and vitamins like vitamin D3 are also examples. Additionally, CβG can solubilize diverse flavonoids with a wide range of applications, including UV-absorbing agents such as umbelliferone used in solar filters, as well as antioxidant and anti-inflammatory substances like curcumin. Different studies have demonstrated that CβG can bind dyes used in medical imaging, potentially reducing their cytotoxicity. Moreover, due to the immunomodulatory properties of CβG, they have been proposed as a new class of adjuvants. CβG have the benefit of being biocompatible and more soluble than cyclodextrins and other synthetic polymers used for drug encapsulation; their negligible cytotoxicity makes them valuable for pharmaceutical applications. In the field of chemistry, CβG can be used in chiral chromatography to separate economically relevant enantiomers. Additionally, they act as catalytic carbohydrates in methanolysis reactions and enhance the efficiency of click reactions by increasing the solubility of highly hydrophobic substrates. Finally, in nanotechnology, CβG have been used to direct the morphology in the synthesis of selenium nanowires (all examples are reviewed in references [5, 8–10]). We expect that the efficient and cost-effective production system of CβG described in this work will promote further research to expand the spectrum of applications of CβG in these and other fields.

When comparing the levels of CβG production of the *E. coli Δmdo*^Cgs^ and *E. coli Δmdo*^Cgs+Cgt^ strains, *E. coli Δmdo*^Cgs+Cgt^ exhibits a higher production of both intracellular and extracellular glucans. This increase in the production of CβG by the *E. coli Δmdo*^Cgs+Cgt^ strain could be attributed to the stabilization of the protein complex Cgs-Cgt and the secretion of CβG to the periplasm by Cgt, and thus avoiding the end-product enzymatic inhibition caused by the accumulation of end products in the cytoplasm. Previous observations have shown that CβG synthesis in permeabilized *Rhizobium* cells is inhibited when the concentration of CβG reached 15 mM (50 g/L) [24], which is close to the estimated concentration of intracellular CβG [25].

In *Brucella*, CβG are produced and remain essentially intracellular. However, in *Agrobacterium* and *Rhizobium* strains CβG can be secreted into the culture medium at different levels depending on the bacterial species and culture conditions [13, 15]. Therefore, we aimed to investigate whether the CβG produced by the *E. coli* engineered strains could be secreted into the extracellular medium, which would be very advantageous for downstream processing. When *E. coli Δmdo*^Cgs^ was cultured in saline medium such as M9, CβG remained intracellular. However, in *E. coli Δmdo*^Cgs+Cgt^ most of the CβG were secreted into the culture medium indicating that CβG would be transported to the periplasm -through the Cgt ABC transporter- before reaching the extracellular space. The mechanism by which CβG are released from the periplasm into the culture medium in *E. coli* remains unknown. Instead, most of the anionic CβG produced by *E. coli Δmdo*^Cgs+Cgt+Cgm^ were retained in the cellular fraction. This location is consistent with the role of anionic periplasmic oligosaccharides in Gram-negative bacteria, which contribute to establish the Donnan potential across the membrane [26, 27]. Similar results were observed for anionic CβG with high DP; however, larger unsubstituted CβG appeared to be retained inside de cells. Recently, Sedzicki et al. observed that, in vitro, there is a preference for transport of CβG with DP of 17 and 18 glucoses and proposed a transport mechanism where the interface of Cgt/CβG mediates the recognition and tight association of some parts of the substrate, while the unbound part of the sugar retains flexibility. Polysaccharides should adopt different conformations to facilitate the docking for subsequent transport, and hence a balance between substrate specificity and size tolerance determines which of the CβG accumulated in the cytoplasm will be transported [17]. The accumulation of higher DP glucans in the bacterial fraction of *E. coli Δmdo* expressing truncated mutants of Cgs is consistent with the mechanism proposed for CβG transport. Recently, the structure of Cgs was resolved by CryoEM [28], confirming all the biochemical information obtained in our lab over the past years and revealing additional insights into the structural and functional complexity of Cgs. It would be interesting to explore the structure of the Cgs-Cgt complex (and Cgs-Cgt-Cgm) using this method to gain insights into the structural factors that determine CβG synthesis, and how it is related with the export and succinylation of these molecules.

It was previously described that the accumulation of high amounts of CβG in the supernatants of *Rhizobium* cultures requires prolonged EFT, as it occurs during the stationary phase under nitrogen-limited conditions when an excess of carbon source is present [13, 16]. Instead in our system, the levels of CβG do not increase when growth is arrested due to nitrogen limitation in the presence of carbon excess (data not shown) and CβG production takes place during active growth. We observed that CβG production follows a similar kinetic pattern to biomass formation, indicating that the amount of CβG should be proportional to the biomass. Based on this correlation, a culture strategy aimed to maximize biomass production by employing HCDC was chosen. This well-established strategy, developed and used in *E. coli* for more than 30 years, is known to achieve high levels of biomass in short EFT, resulting in high productivities. Furthermore, HCDC is performed using a cost-effective and safe saline medium [23, 29] which offers an additional advantage over complex media by simplifying CβG purification from the culture supernatant. Using this technology, we obtained a yield of 4.5 g of pure CβG L^− 1^ in 48 h in culture supernatant, equivalent to a productivity of 94 mg CβG L^− 1^h^− 1^, the higher productivity reported to date [13].

The new recombinant CBG production system described here represents an improvement in productivity over known systems. It enables the achievements of comparable CBG yields in 75% less fermentation time, thereby reducing labor costs and energy consumption. This translates to more efficient utilization of installed capacity. Furthermore, the carbon source utilized is glycerol, an abundant byproduct of biofuel manufacturing, rendering the process economically viable and environmentally sustainable.

Finally, one of the system’s strengths lies in the possibility of further optimization of productivity. In this regard, efforts should be directed at optimizing the host strain to identify and address any bottlenecks in glucan production. This may involve the fine-tuning of Cgs and Cgt expression and/or the enhancement of substrate availability. Adjusting culture conditions, such as medium composition, feeding rate, and temperature, could also prove beneficial. Furthermore, current purification protocols for high volumes involve two sequential ethanol precipitation steps (75% and 90%, with overnight cold incubation) to separate long-chain polysaccharides from CβG, followed by SEC chromatography. To improve the downstream process, it would be critical to determine the composition of CβG crude extract in our system, with the objective of developing more efficient and scalable purification.

## Conclusions

We have developed a new recombinant bacterial system for the synthesis of cyclic β-1,2-glucans, expanding the available toolbox for the production of glycomolecules. The combination of this new approach with the use of the HCDC technology allowed us to produce CβG in *E. coli* with high yields and the highest volumetric productivity reported to date. We expect this new highly scalable system facilitates CβG availability for further research and the widespread use of these promising molecules across many application fields.

## Methods

### Bacterial strains and plasmids

Construction of the *E. coli Δmdo* strain and pCgsHT, pCgt-3xFlag and pUT18Cgm plasmids was previously described [18].

### Culture media and growth conditions

Initial cultures to characterize the production and secretion of CβG by the *E. coli Δmdo*^Cgs^ (expressing the *B. abortus* CβG synthase (Cgs)), *E. coli Δmdo*^Cgs+Cgt^ (expressing Cgs and the CβG transporter (Cgt)), and *E. coli Δmdo*^Cgs+Cgt+Cgm^ (expressing Cgs, Cgt and the CβG modifier Cgm) strains were performed in M9 medium with 10 g/L glycerol as carbon source. For shake flask (Erlenmeyer) and bioreactor assays, we used Korz medium with 10 g/L glycerol as the carbon source unless another concentration was specified. Korz medium was prepared as described by Korz et al. [23], except that 0.2 mM CaCl_2_ was added to the culture medium prior inoculation, and the feeding solution was: 300 g/l Glycerol, 20 g/L MgSO_4_.7H_2_O, 40 g/L (NH_4_)_2_SO_4_, 0.1 g Fe(III)citrate, 0.0225 g MnCl_2_.4H_2_O, 0.017 g ZnSO_4_.7H_2_O, 0.005 g H_3_BO_3_, 0.004 g Na_2_MoO_4_.2H_2_O and 0.004 g CoCl_2_.6H_2_O. Shake flask experiments were incubated in a rotary shaker at 200 rpm. All cultures were incubated at 30 °C. Media were supplemented with antibiotics at the following concentrations: kanamycin 50 µg/ml and tetracycline 2.5 µg/ml.

### Precultures

A few colonies of *E. coli Δmdo*^Cgs+Cgt^ were transferred from LB agar plates to 10 ml of Korz medium and incubated in a rotary shaker at 30 °C and 200 rpm for 20–24 h to generate the seed culture 1. For shake flask experiments, the seed culture 1 was used to inoculate 50 ml Erlenmeyers with 10 ml of Korz medium at an initial OD_600_ of around 0.15. For bioreactor experiments, seed culture 1 was used to inoculate the seed culture 2 consisting of 300 ml of Korz medium in a 4 L Erlenmeyer. The seed culture 2 was incubated on a rotary shaker at 30 °C for 20–24 h and used to inoculate the bioreactor at the desired OD_600nm_.

### Bioreactor cultures

Bioreactor cultures were performed in a 7-L BioFlo 110 stirred tank bioreactor (New Brunswick Scientific, Edison, NJ) connected to the Biocommand software (New Brunswick Scientific) for parameter monitoring and control. The temperature was set at 30 °C and the aeration with compressed air was manually adjusted between 0.5 and 1.5 vessel volumes per minute (vvm). The impeller speed was adjusted between 300 and 1200 rpm, and dissolved oxygen was registered on-line with a polarographic probe. The pH was maintained at 6.70 by addition of H_3_PO_4_ (28%) or NH_4_OH (25%) and controlled on-line with a pH electrode. A sterile solution of Antifoam 204 0.3% (Sigma) was automatically added to control the foam level. During the process, samples were taken for measuring the optical density at 600 nm and the supernatant was stored at -20 °C for subsequent determination of total reducing sugars (TRS) or CβG purification and characterization.

### CβG extraction and TLC analysis

Bacterial cultures were harvested by centrifugation. For cellular CβG analysis, CβG were extracted from cell pellets with 75% ethanol. Briefly, the pellets were resuspended in 0.1 to 1 volume of 75% ethanol and incubated at 37 °C for 1 h. Cells were then removed by centrifugation, the ethanolic extracts were dried in a Speed-Vac centrifuge and the extracted CβG were resuspended in 15 µl of 70% ethanol. Thin Layer Chromatography (TLC) was performed on silica gel 60 plates (Merck). For characterization of extracellular CβG, 1 ml of each culture supernatant was desalted using PD-10 columns (Cytiva), eluted with Milli-Q® water and 10% of the eluted volumes were dried in a Speed-Vac centrifuge. Dried fractions of CβG were resuspended in 15 µl of 70% ethanol and subjected to TLC analysis on Silica Gel 60 plates (Merck). For TLC characterization of extracellular CβG in high cell density bioreactor cultures, CβG were purified by 90% ethanol precipitation. Briefly, 3 ml of ethanol were added to 1 ml of culture supernatant and centrifuged for 30 min at 4 °C and 4500 g; then, CβG in the supernatant were precipitated adding 6 ml of ethanol (90% ethanol final) and incubated overnight at 4 °C. CβG were separated by centrifugation, resuspended in 1 ml of Milli-Q® water and a sample of 5 µl was subjected to TLC analysis. In all cases, the TLC plates were run 2 or 3 times with 5:5:4 ethanol: butanol: water and developed with 5% sulfuric acid solution in ethanol and heating at 125 °C for 12 min, as previously described [11].

### Determination of total reducing sugars by the anthrone-sulfuric acid method

To quantify the total reducing sugars (TRS), a microplate adapted version of the anthrone-sulfuric acid method [30] was used. Briefly, 133 µl of anthrone solution (2 g/L anthrone in sulfuric acid) were added to 67 µl of each sample or the standard and mixed up and down with the micropipette in 96-well plates. The plates were heated to 95 °C for 10 min and allowed to cool for 10 min. Absorbance was measured in a microplate reader with a 630 nm filter. A glucose standard curve (15 to 350 µg/ml) was used for quantification. An internal standard of known concentration of CβG was included in each measurement to verify that the polysaccharide hydrolysis was complete. Results for CβG concentration were expressed as mg of glucose equivalents per L.

### Size exclusion chromatography

CβG were purified by size exclusion chromatography (SEC) to separate CβG from other oligo- and monosaccharides. Briefly, 300 µl of culture supernatant was applied to the column (16/170 mm, BiogelP6, BioRad) using water as mobile phase at 0.15 ml/min. Fractions of 0.5 ml were collected and TRS were quantified by the anthrone-sulfuric acid method. CβG concentration was calculated from the fractions corresponding to the CβG peak. The identity of each peak component was confirmed by TLC analysis.

### Matrix-assisted laser desorption/ionization-time of flight-mass spectrometry

Matrix-Assisted Laser Desorption/Ionization-Time of Flight-Mass Spectrometry (MALDI-TOF-MS) of CβG produced in Erlenmeyer was performed using an Ultraflex II MALDI-TOF/TOF mass spectrometer equipped with an ultraviolet high-performance solid-state laser (λ = 355 nm) and a reflector. The system was operated by Flexcontrol 3.3 software package (Bruker Daltonics GmbsH, Germany). The recorded spectra were the result of 1000–1500 laser shots. All samples were measured in the linear and reflectron mode, and as routine in both positive and negative polarity. Laser power was typically 40–60% of its maximum intensity and the accelerating voltage 20 kV.

External calibration was carried out using the commercial proteins bradykinin 1–7 (MW 757.399), angiotensin I (MW 1296.685), renin substrate (MW 1758.933) and insulin β-chain (MW 3494.6506) with CHCA (alpha-cyano-4-hydroxy-cinnamic acid) matrix, and β-cyclodextrin (cycloheptaamylose, MW1135.0) and γ-cyclodextrin (cyclooctaamylose, MW 1297.1) with norharmane matrix. The samples were loaded onto a ground steel sample plate (MTP 384 ground steel; Bruker Daltonics GmbsH) using the sandwich method.

MALDI-TOF-MS of CβG produced in Bioreactor was performed using a Microflex MALDI-TOF mass spectrometer. External calibration was performed with angiotensin I (MW1296.685), substance P (MW 1347.7354), bombesin (MW 1619.822), ACTH dip 1–17 (MW 2093.086), ACTH dip 18–39 (MW 2465.198), Somatostatin 28 (MW 3147.471) with CHCA matrix in linear mode and positive polarity.

### Electronic supplementary material

Below is the link to the electronic supplementary material.

**Supplementary Material 1**: **Fig. S1**. (A) Growth rate and (B) biomass yield of *E. coli Δmdo*^Cgs+Cgt^ as a function of substrate concentration. The kinetic and stoichiometric parameters obtained in Erlenmeyer were used lately to design and calculate the bioreactor culture strategy. The results are representative of two independent experiments. Error bars indicate the standard deviation.

## Data Availability

The dataset supporting the conclusions of this article are all included in the article.

## Abbreviations

CβG: Cyclic β-1,2-glucans
HCDC: High Cell Density Culture
Cys: Cyclosophoraoses
DP: Degree of polymerization
Cgs: CβG synthase
Cgt: CβG transporter
Cgm: CβG modifier
OPGs: Osmoregulated periplasmic glucans
TLC: Thin Layer Chromatography
TRS: Total reducing sugars
SEC: Size exclusion chromatography
EFT: Effective fermentation time
CHCA: Alpha-cyano-4-hydroxy-cinnamic acid

## References

[1] Moradali MF, Rehm BHA (2020). Bacterial biopolymers: from pathogenesis to advanced materials. Nat Rev Microbiol.

[2] Venkatachalam G, Arumugam S, Doble M (2021). Industrial production and applications of α/β linear and branched glucans. Indian Chem Eng.

[3] Yates LE, Mills DC, DeLisa MP, Rapp E, Reichl U (2018). Bacterial glycoengineering as a Biosynthetic Route to customized glycomolecules. Advances in Glycobiotechnology.

[4] Breedveld MW, Miller KJ (1994). Cyclic beta-glucans of members of the family Rhizobiaceae. Microbiol Rev.

[5] Guidolin LS, Arce-Gorvel V, Ciocchini AE, Comerci DJ, Gorvel JP (2018). Cyclic beta-glucans at the bacteria-host cells interphase: one sugar ring to rule them all. Cell Microbiol.

[6] Braga SS, Cyclodextrins. Emerging Medicines of the New Millennium. Biomolecules. 2019;9(12). 10.3390/biom9120801 PMC6995511 31795222

[7] Braga SS. Molecular Mind Games: The Medicinal Action of Cyclodextrins in Neurodegenerative Diseases. 2023;13(4):666. 10.3390/biom13040666 PMC10136110 37189413

[8] Cho E, Jeong D, Choi Y, Jung S (2016). Properties and current applications of bacterial cyclic β-glucans and their derivatives. J Incl Phenom Macrocyclic Chem.

[9] Venkatachalam G, Nandakumar V, Suresh G, Doble M (2014). Characterization and applications of cyclic β-(1,2)-glucan produced from R. meliloti. RSC Adv.

[10] Martirosyan A, Pérez-Gutierrez C, Banchereau R, Dutartre H, Lecine P, Dullaers M (2012). Brucella β 1,2 cyclic glucan is an activator of human and mouse dendritic cells. PLoS Pathog.

[11] Iñón de Iannino N, Briones G, Tolmasky M, Ugalde RA (1998). Molecular cloning and characterization of cgs, the Brucella abortus cyclic beta(1–2) glucan synthetase gene: genetic complementation of Rhizobium meliloti ndvB and Agrobacterium tumefaciens chvB mutants. J Bacteriol.

[12] McIntire FC, Peterson WH, Riker AJ (1942). A polysaccharide produced by the crown-gall organism. J Biol Chem.

[13] Venkatachalam G, Srinivasan D, Doble M (2013). Cyclic β-(1, 2)-glucan production by Rhizobium meliloti MTCC 3402. Process Biochem.

[14] Zevenhuizen LP, Scholten-Koerselman HJ (1979). Surface carbohydrates of Rhizobium. I. Beta-1, 2-glucans. Antonie Van Leeuwenhoek.

[15] Hisamatsu M (1992). Cyclic (1–2)-beta-D-glucans (cyclosophorans) produced by Agrobacterium and Rhizobium species. Carbohydr Res.

[16] Breedveld MW, Zevenhuizen LP, Zehnder AJ (1990). Excessive excretion of cyclic beta-(1,2)-glucan by Rhizobium trifolii TA-1. Appl Environ Microbiol.

[17] Sedzicki J, Ni D, Lehmann F, Wu N, Zenobi R, Jung S (2022). Mechanism of cyclic β-glucan export by ABC transporter Cgt of Brucella. Nat Struct Mol Biol.

[18] Guidolin LS, Morrone Seijo SM, Guaimas FF, Comerci DJ, Ciocchini AE (2015). Interaction network and localization of Brucella abortus membrane proteins involved in the synthesis, transport, and succinylation of cyclic beta-1,2-glucans. J Bacteriol.

[19] Ciocchini AE, Guidolin LS, Casabuono AC, Couto AS, de Iannino NI, Ugalde RA (2007). A glycosyltransferase with a length-controlling activity as a mechanism to regulate the size of polysaccharides. Proc Natl Acad Sci U S A.

[20] Guidolin LS, Ciocchini AE, Inon de Iannino N, Ugalde RA (2009). Functional mapping of Brucella abortus cyclic beta-1,2-glucan synthase: identification of the protein domain required for cyclization. J Bacteriol.

[21] Roset MS, Ciocchini AE, Ugalde RA, Inon de Iannino N (2004). Molecular cloning and characterization of cgt, the Brucella abortus cyclic beta-1,2-glucan transporter gene, and its role in virulence. Infect Immun.

[22] Roset MS, Ciocchini AE, Ugalde RA, Inon de Iannino N (2006). The Brucella abortus cyclic beta-1,2-glucan virulence factor is substituted with O-ester-linked succinyl residues. J Bacteriol.

[23] Korz DJ, Rinas U, Hellmuth K, Sanders EA, Deckwer WD (1995). Simple fed-batch technique for high cell density cultivation of Escherichia coli. J Biotechnol.

[24] Breedveld MW, Zevenhuizen LP, Zehnder AJ (1992). Synthesis of cyclic beta-(1,2)-glucans by Rhizobium leguminosarum biovar trifolii TA-1: factors influencing excretion. J Bacteriol.

[25] Arellano-Reynoso B, Lapaque N, Salcedo S, Briones G, Ciocchini AE, Ugalde R (2005). Cyclic beta-1,2-glucan is a Brucella virulence factor required for intracellular survival. Nat Immunol.

[26] Miller KJ, Kennedy EP, Reinhold VN. Osmotic adaptation by Gram-negative Bacteria: possible role for Periplasmic Oligosaccharides. 1986;231(4733):48–51. 10.1126/science.3941890 3941890

[27] Bontemps-Gallo S, Bohin JP, Lacroix JM. Osmoregulated Periplasmic Glucans EcoSal Plus. 2017;7(2). 10.1128/ecosalplus.esp-0001-2017 PMC11575687 28593831

[28] Sedzicki J, Ni D, Lehmann F, Stahlberg H, Dehio C (2024). Structure-function analysis of the cyclic β-1,2-glucan synthase from Agrobacterium tumefaciens. Nat Commun.

[29] Shiloach J, Fass R, Growing E (2005). Coli to high cell density: a historical perspective on method development. Biotechnol Adv.

[30] Leyva A, Quintana A, Sánchez M, Rodí­guez EN, Cremata J, Sánchez JC (2008). Rapid and sensitive anthrone-sulfuric acid assay in microplate format to quantify carbohydrate in biopharmaceutical products: method development and validation. Biologicals.

