# Supplementary material for: Development of a scalable recombinant system for cyclic beta-1,2-glucans production
Source: Microb Cell Fact. 2024 May 6;23:130. doi: 10.1186/s12934-024-02407-z (PMC11071196; doi:10.1186/s12934-024-02407-z)
Supplement: Supplementary file 1 — Supplementary Material 1: Fig. S1. (A) Growth rate and (B) biomass yield of E. coli ΔmdoCgs+Cgt as a function of substrate concentration. The kinetic and stoichiometric parameters obtained in Erlenmeyer were used lately to design and calculate the bioreactor culture strategy. The results are representative of two independent experiments. Error bars indicate the standard deviation. [file 12934_2024_2407_MOESM1_ESM.pdf]

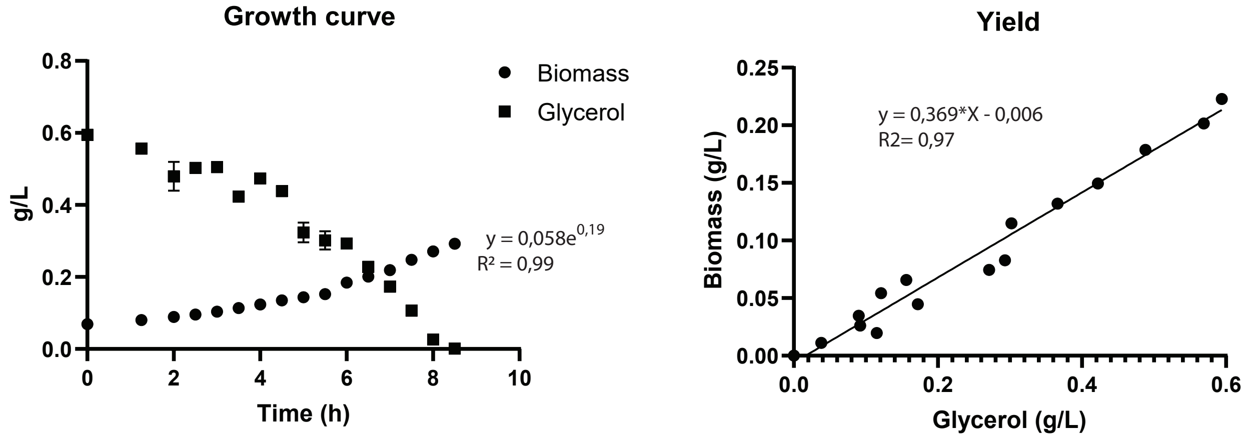

Figure S1

Figure S1: (A) Growth rate and (B) biomass yield of E. coli $\Delta mdo^{Cgs+Cgt}$ as a function of substrate concentration. The kinetic and stoichiometric parameters obtained in Erlenmeyer were used lately to design and calculate the bioreactor culture strategy. The results are representative of two independent experiments. Error bars indicate the standard deviation.
